# Research on highway rain monitoring based on rain monitoring coefficient

**DOI:** 10.1038/s41598-024-53360-1

**Published:** 2024-02-23

**Authors:** Xingyu Wang, Haixia Feng, Na Wang, Maoxin Zhu, Erwei Ning, Jian Li

**Affiliations:** 1 School of Transportation and Logistics Engineering, Shandong Jiaotong University, Jinan, 250023 China; 2 Shandong Intelligent Transportation Key Laboratory (Preparatory), Jinan, 250023 China

**Keywords:** Engineering, Physics

## Abstract

The real-time and accurate monitoring of severe weather is the key to reducing traffic accidents on highways. Currently, rainy day monitoring based on video images focuses on removing the impact of rain. This article aims to build a monitoring model for rainy days and rainfall intensity to achieve precise monitoring of rainy days on highways. This paper introduces an algorithm that combines the frequency domain and spatial domain, thresholding, and morphology. It incorporates high-pass filtering, full-domain value segmentation, the OTSU method (the maximum inter-class difference method), mask processing, and morphological opening for denoising. The algorithm is designed to build the rain coefficient model P_rain coefficient_ and determine the intensity of rainfall based on the value of P_rain coefficient_. To validate the model, data from sunny, cloudy, and rainy days in different sections and time periods of the Jinan Bypass G2001 line were used. The aim is to raise awareness about driving safety on highways. The main findings are: the rain coefficient model P_rain coefficient_ can accurately identify cloudy and rainy days and assess the intensity of rainfall. This method is not only suitable for highways but also for ordinary road sections. The model's accuracy has been verified, and the algorithm in this study has the highest accuracy. This research is crucial for road traffic safety, particularly during bad weather such as rain.

## Introduction

By the end of 2022, China had achieved a high-speed mileage of 177,000 km, with the national motor vehicle ownership exceeding 417 million units. The highway mileage and the number of motor vehicles are steadily ranked first in the world. Although the trend of traffic accidents and the death rate per 10,000 vehicles have been decreasing year by year, the rate remains high, and the death rate on highways is significantly higher than that on ordinary roads. According to statistics from the Public Security Bureau of the Ministry of Communications, the death rate on highways in China is 4.51 times higher than that on ordinary highways. Accidents caused by changes in visibility due to rainy, snow, fog, and other bad weather account for approximately 45% of all traffic accidents.

The highway has narrow width, long mileage, large span, and difficulty in real-time monitoring and early warning due to the changeable weather conditions. It is difficult to cover the entire highway with micro meteorological stations due to their high instrument prices. Therefore, research on the monitoring model of severe weather based on video images has become a research hotspot. At present, there are more studies on the monitoring of fog, visibility, and snow based on video images. Gunawan et al. ^1^ use the classic AlexNet to detect visibility information from foggy images, thereby judging the visibility; Tang et al. ^2^ use an improved VGG16 network to extract image features, and accurately identify the fog weather visibility level of the highway; Ismail ^3^, Zhou ^4^ et al. use single-scale and multi-scale Retinex algorithms. They eliminate the interference of uneven light in foggy images according to the color of objects being irrelevant to light and illumination changes. Elhashemi et al. ^5^ use trajectory-level data extracted from the Strategic Highway Research Program (SHRP2) Natural Driving Study (NDS) dataset to detect real-time snow weather conditions on highways. Quan et al. ^6^ use a reversible neural network (INN) to propose a single image snow removal method based on deep learning. They explain snow removal as an image decomposition problem and can accurately recognize snowflakes. Chiu et al. ^7^ propose a lightweight residual network that can significantly enhance snowy images. Lv et al. ^8^ construct a road segmentation fusion network that combines global features of road weather images with road features to achieve recognition of severe weather such as snow days. However, there is little research on rain detection and early warning, and more research focuses on how to remove the impact of rain to improve vehicle and license plate monitoring accuracy. Barnum et al. ^9^ analyze the physical and spatio-temporal statistical characteristics of raindrops, combine a precise stripe model with spatio-temporal statistical characteristics of raindrops, and construct a dynamic weather model in the frequency space based on this model to achieve rain removal. Jin et al. ^10^ propose an asynchronous interactive generative adversarial network (AI-GAN) to achieve complementary adversarial optimization, avoid over-smoothing of local regions in the restored image. Kang et al. ^11^ use image decomposition to eliminate rainfall by dictionary learning and sparse coding, eliminating the rain component in single images. Hu et al. ^12^ use a deep unfolding network (DUN) combined with proximal gradient descent (PGD) algorithms for single image rain removal. Thatikonda et al. ^13^ propose a transformer DeTformer for rain removal that can effectively utilize local image features to make the effect of rain removal more obvious. Fu et al. ^14^ mimic brain synaptic plasticity mechanisms in learning and memory processes to effectively alleviate forgetting and enable a single network to process multiple datasets, enhancing the effect of rain removal. However, rain removal and rainy day monitoring and recognition are two different tasks. Image rain removal is an image enhancement task that adjusts the value of each pixel to improve image quality, while rainy day recognition is based on analyzing the features of images to judge and monitor rainy days. Therefore, it is extremely important to develop a suitable algorithm and system for rainy day monitoring.

In summary, with the development of artificial intelligence in recent years, most researchers have used machine learning or deep learning methods for severe weather research. Although this method is popular, there are still some defects because it is still in its developing stage. For example, it cannot accurately judge rainy days, and the computational requirements are high for hardware requirements. Based on the above problems, this article returns to traditional algorithms and strives to obtain accurate results with minimal cost. Inspired by Ji ^15^, Zhang ^16^, Deng ^17^, Otsu's method, this article proposes an algorithm based on combining frequency domain and spatial domain, threshold and morphology, to achieve monitoring of rainy weather. Otsu's method is mostly used in agricultural ^18,19^, medical ^20^, water conservancy ^21–23^ and other fields, but current research has not yet been applied to highway rainy day recognition. This article is based on video image data of highways. Through Otsu's method, high-pass filtering, gray feature values, whole domain segmentation, mask processing and other methods, this article extracts the road by binarizing the image and extracting the part that is covered by accumulated water due to rain on the road (indicated by white pixel blocks). Through morphological opening operation ^24^, the influence of noise is eliminated, and finally a rain coefficient model is constructed to determine whether it is a rainy day and to judge the intensity of the rain.

## Materials and methods

### Data

#### Study area

**Figure 1:**
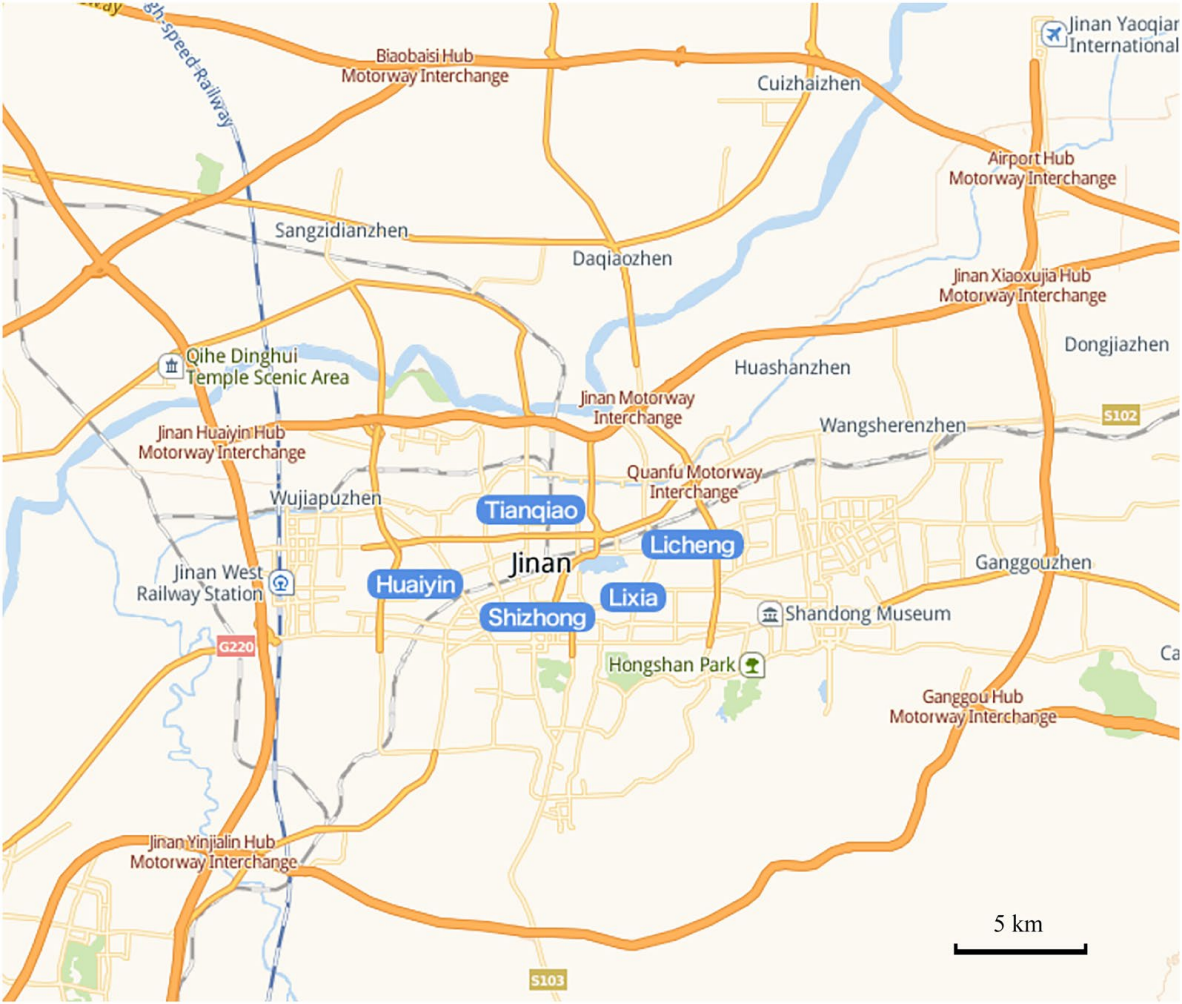
Jinan Bypass Highway G2001 Line. The above picture shows the Jinan Bypass Highway G2001 Line.

The selected study area is the area captured by the camera at K15+0.10 km on Jinan Bypass Highway G2001, which is a bypass highway that links the main national highways—Beijing-Shanghai, Beijing-Fuzhou, and Qingdao-Yinchuan—at the periphery of Jinan, the capital of Shandong Province. As can be seen in Fig. 1, this area has a unique transportation system that serves as a crucial link in the regional transportation network.

#### Field monitoring data

The video data used in this article were collected from high-definition video surveillance equipment installed at the meteorological observatory in Jinan. The data collection period was from September to October 2022. Three common weather conditions, namely, cloudy, sunny, and rainy days, were selected, and three experimental videos were recorded during the selected periods. The corresponding weather conditions for the video sections during these periods were also obtained.

### Methods

#### Image feature analysis of different weather conditions

**Figure 2:**
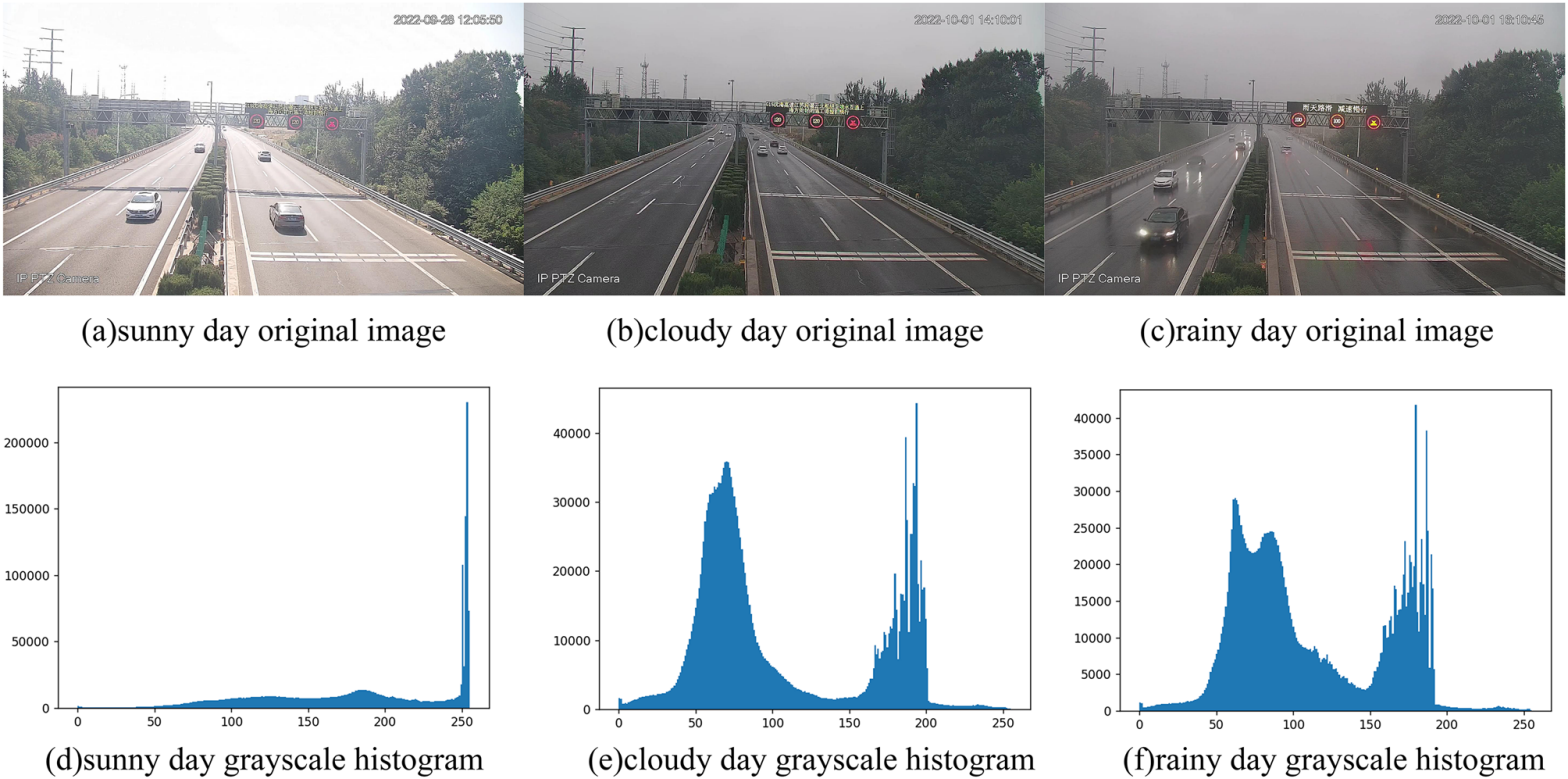
Original image and grayscale histogram on sunny, cloudy, and rainy days. The above image shows the original image and grayscale histogram of three types of weather: sunny, cloudy, and rainy.

Firstly, the grayscale histograms of the images of sunny, cloudy, and rainy days were analyzed, as shown in Fig. 2.

It can be seen from Fig. 2d–f that the grayscale histogram of sunny days is relatively stable compared to cloudy and rainy days. The color saturation of sunny images is high, which is reflected in the grayscale image as a distinct single-peak image. Moreover, under clear weather conditions, a significant peak is formed around grayscale value 250, which is due to the significantly higher grayscale values in sunny days compared to cloudy and rainy days. The histograms of cloudy and rainy days both exhibit a clear double-peak phenomenon, as shown in Fig. 2e,f. The first peak represents the peak of the road section, while the second peak represents the peak of the sky section. However, in rainy days, there is also a small peak phenomenon, as shown in Fig. 2f, due to the influence of rain, which causes water accumulation on the road section, leading to an increase in image saturation.

Since the grayscale histograms of cloudy and rainy days are similar, this article constructs a rainy day monitoring model based on the grayscale histograms of cloudy and rainy days to effectively distinguish between cloudy, light rainy, moderate rainy, and heavy rainy weather in real-time.

#### Principles of model construction

**Figure 3:**
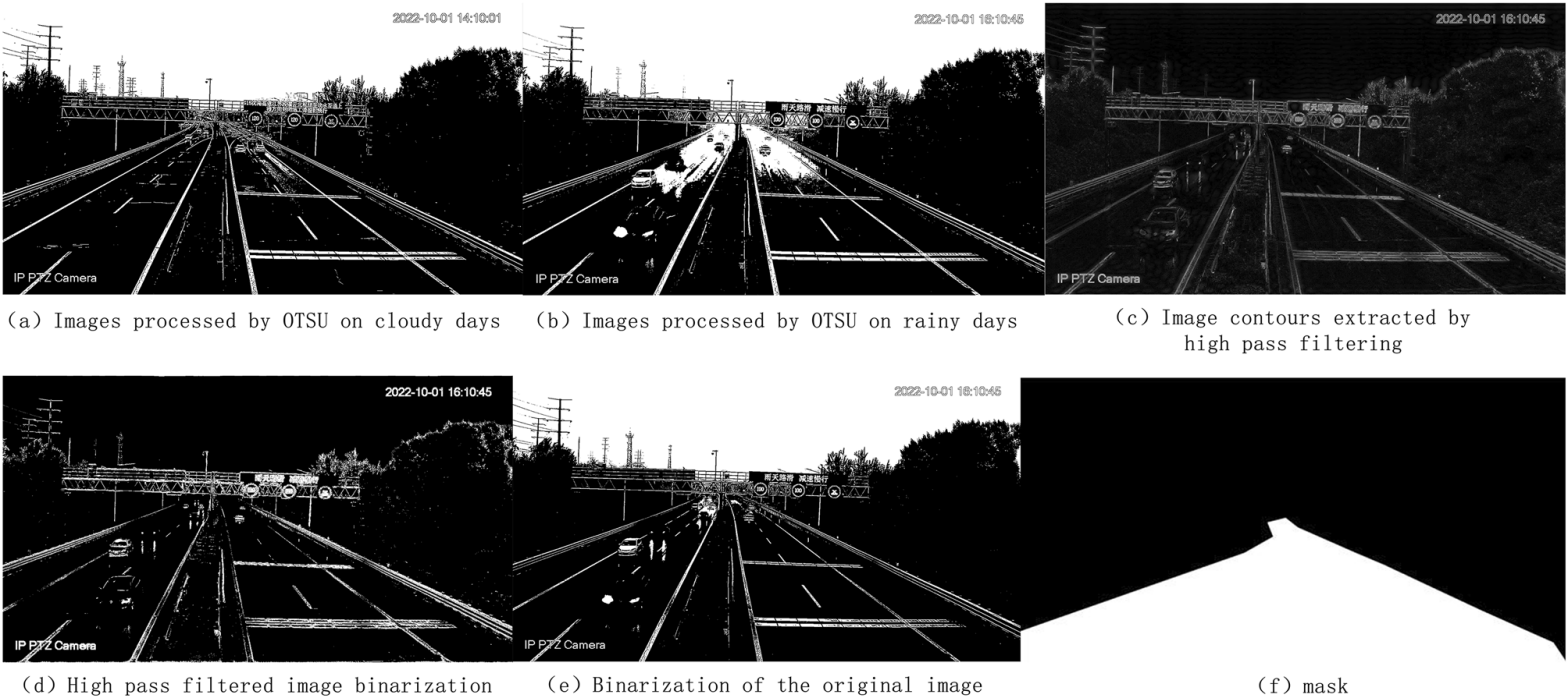
Schematic diagram of model construction. The above figure shows the principles required for constructing a rain monitoring model.

The rainy day monitoring model constructed in this article uses the following principles (this section only introduces the principles of construction, and the specific formulas used in the principles are presented in section "Construction of road rainy day monitoring coefficient model"):

(1) OTSU method: This is an image grayscale adaptive threshold segmentation algorithm that can automatically determine the threshold based on image differences. This method can effectively distinguish between cloudy and rainy days. The binary images obtained by applying the OTSU method to cloudy and rainy days are shown in Fig. 3a,b.

As shown in Fig. 3, the images processed by the OTSU method have distinct differences in image features. The construction principle of the rainy day monitoring model is to calculate the proportion of white pixel blocks in the road section of the image to the total pixel blocks of the road, thereby determining the weather conditions as cloudy, light rainy, moderate rainy, or heavy rainy. However, when observing the road section of the image, it can be seen that high-grayscale vehicles and road lines also become white after OTSU processing. To improve the calculation accuracy, it is necessary to remove the high-grayscale vehicles and road lines.

(2) High-pass filtering: This uses high-pass filtering to extract contours from the image, removing road lines and vehicle contours. In the frequency domain, low-frequency regions have small grayscale values and the image is smooth, while high-frequency regions have large grayscale values and the image is rough, often representing edges or noise. The purpose of using high-pass filtering is to highlight edges and retain the portions with higher frequencies. Figure 3c shows an example of rainy day high-pass filtering.

(3) Global threshold segmentation: This method specifies a threshold based on the grayscale histogram. The rainy day monitoring model construction uses global threshold segmentation twice. The first time is to segment the road lines and vehicle contours extracted by high-pass filtering, binarizing the image, as shown in Fig. 3d. The second time is to segment the original image using global threshold segmentation, setting the threshold to 150 (as shown in Fig. 2f., 150 is at the trough of the grayscale histogram, and the grayscale values greater than 150 will be classified as white, thereby extracting high-grayscale vehicles. Since the water on the road surface is less than 150, it will not be extracted), as shown in Fig. 3e.

(4) Masking: To improve model accuracy, we selected the road as the study area. However, there are also white areas, so we masked and extracted the road section as shown in Fig. 3f.

(5) Morphological denoising: Morphological operations are typically used on binary images for boundary extraction, skeleton extraction, hole filling, corner detection, and image reconstruction. Basic algorithms include dilation and erosion, opening and closing operations. Erosion and dilation are the basis of morphological operations. In practical detection processes, combinations of erosion and dilation are often used to process images. To reduce noise effects, morphological opening operation (erosion followed by dilation) denoising treatment was applied at the end of the model. Erosion can remove small noise points on the image, but while removing noise, it can also affect other parts of the image. Dilation can reduce the impact of dilation.

#### Construction of road rainy day monitoring coefficient model

Based on the principle described in 1.2.2, a rainy day monitoring coefficient model was constructed to monitor road rainy days, as shown in formula (1).

1 $$ P_{{{\text{rainy}}\,{\text{coefficient}}}} = \frac{{[(OM_{i,j} - GQ_{1} M_{i,j} - Q_{2} M_{i,j} )\Theta B] \oplus B}}{{IM_{i,j} }} $$

where the P_rain coefficient_ is the percentage of white pixel blocks.

O represents the image processed by the Otsu method; G represents the image after high-pass filtering; Q_1_ represents the image obtained by performing full threshold segmentation and binarization on the high-pass filtered image; Q_2_ represents the image obtained by performing full threshold segmentation and binarization on the original image; M_i,j_ represents the mask, M_i,j_=0 or 1. The image range covered by 0 is removed, and the image range covered by 1 is retained. Represents the subtraction operation on the three masked binary images. B represents the structural element for morphological processing, which is used for image erosion and dilation; Θ represents the corrosion operation; ⊕ indicates the inflation operation; I represents the original image.

Each of these four methods is described below.

(1) OTSU method

The calculation method of the OTSU method is shown in the following formula:

2 $$ g = \omega_{0} (\mu_{0} - \mu )^{2} + \omega_{1} (\mu_{1} - \mu )^{2} $$

where g is the interclass variance.

ω_0_ is the number of pixel points in the foreground (black) as a proportion of the whole image, the

3 $$ \omega_{0} = \frac{{N_{0} }}{M*N} $$

ω_1_ is the ratio of the number of pixel points in the background (white) to the whole image,

4 $$ \omega_{1} = \frac{{N_{1} }}{M*N}; $$

The size of the image is M*N.

The number of pixels in the image with grayscale values less than the threshold is denoted as N_0_, and the number of pixels with grayscale values less than the threshold is denoted as N. The number of pixels whose grayscale is greater than the threshold is denoted as N_1_. μ_0_ is the average gray level of the pixel points in the foreground (black). μ_1_ is the average gray level of the pixel points in the background (white). μ is the total average gray level of the image, the

5 $$ \mu = \omega_{0} *\mu_{0} + \omega_{1} *\mu_{1} $$

Bringing the total average grayness into the formula for the between-class variance, we obtain the equivalent formula, the

6 $$ g = \omega_{0} \omega_{1} (\mu_{0} - \mu_{1} )^{2} . $$

The threshold T, which maximizes the variance g between classes, is obtained using the traversal method, which is the desired threshold result.

(2) High-pass filtering

The purpose of high-pass filtering of images is to obtain edge features, which in this paper is used to obtain the contours of road routes and vehicles to remove their effects. In the frequency domain, the low-frequency domain where the gray value is small image is smooth, while in the high-frequency domain where the gray value is large and the image is rough, often edges or noise. The purpose of using high-pass filtering is to highlight the edges and retain the parts with higher frequencies. In this paper, high-pass filtering is used to achieve the purpose of extracting vehicle edges and removing vehicle effects.

Firstly, the image details are enhanced by applying the Fourier positive inverse transform, combined with a high-pass filter to filter out the low frequencies and retain only the high frequencies. Discrete Fourier Transform (DFT) can transform the continuous spectrum into a discrete spectrum to calculate. DFT is defined as letting x (n) be a finite length sequence of length M, then defining the N-point discrete Fourier transform of x ( n ) as

7 $$ {\text{X(k)}} = {\text{DFT[X(n)]}} = \sum\limits_{{{\text{n}} = 0}}^{{{\text{N}} - 1}} {{\text{X(n)W}}_{{\text{N}}}^{{{\text{kn}}}} } (k = 0,1, \ldots ,N - 1) $$

Discrete Fourier Inverse Transform (IDFT): The Discrete Fourier Inverse Transform (IDFT) of x (k) is

8 $$ {\text{X(n)}} = I{\text{DFT[X(k)]}} = \frac{1}{N}\sum\limits_{{{\text{n}} = 0}}^{{{\text{N}} - 1}} {{\text{X(k)W}}_{{\text{N}}}^{{ - {\text{kn}}}} } \,(n = 0,1, \ldots ,N - 1) $$

$W_{N}^{kn}$ is the DFT matrix, $$W_{N}^{kn} = \left[ {\begin{array}{*{20}c} {W_{N}^{0*0} } & {W_{N}^{0*1} } & \cdots & {W_{N}^{0*n} } \\ {W_{N}^{1*0} } & {W_{N}^{1*1} } & \cdots & {W_{N}^{1*n} } \\ \cdots & {} & \cdots & {} \\ {W_{N}^{k*0} } & {W_{N}^{k*1} } & \cdots & {W_{N}^{k*n} } \\ \end{array} } \right]$$, and N is called the length of the DFT transform interval, N ≥ M.

9 $$ {\text{Output}}\,{\text{image}}\quad G = I*( - 1)^{x + y} *X(k)*H(u,v)*X(n)*( - 1)^{x + y} $$

where G is the high-pass filtered image; I is the original image; (− 1)^x+y^ is the centering of the spectrum, where the high-frequency signals are concentrated in the center; X(k) is the conversion of the image into frequencies by discrete Fourier transform; H(u,v) is the high-pass filter, where some frequencies can be suppressed or enhanced while others remain unchanged; X(n) is the inverse transformation of the processed spectrum; after the above processing, the image is obtained by multiplying (− 1) by the first step to obtain the high-pass filtered image. After the above processing, the image of the spectrum centered after the first step is multiplied by (− 1)^x+y^ and finally multiplied by (− 1)^x+y^ to obtain the image after high-pass filtering.

The images obtained after high-pass filtering are binarized and used to construct a rain coefficient model.

(3) Full domain value segmentation

Full domain value segmentation process to remove the influence of vehicles with high gray value, grayscale processing of the original image, and get the gray histogram, from the gray histogram to select the appropriate threshold value, the threshold value is selected as the location of the valley in the bimodal map, such as 2.2.1 construct the principle of the gray histogram of the rainy day, and then the image for the full domain value segmentation, to get the full domain value segmentation binarization image Q_2_.

Image Binarization is the process of converting the pixel points of an image grayscale value is set to 0 or 255, and the full-field value segmentation image binarization formula is as follows.

10 $$ dst(x,y) = \left\{ {\begin{array}{*{20}l} {\max {\mkern 1mu} V{\mkern 1mu} AL} \hfill & {\quad src(x,y) \ge thread} \hfill \\ 0 \hfill & {\quad othersize} \hfill \\ \end{array} } \right. $$

(4) mask

The mask is used to extract the parts of interest in the image and mask out the parts that are not of interest. Each pixel in the original image and each corresponding pixel in the mask is compared with the operation: 1 & 1 = 1; 1 & 0 = 0.

(5) Morphological processing

This article uses the open operation, which involves etching followed by expansion, to remove the influence of noise points on the highway. The open operation formula is as follows

11 $$ A \circ B = (A\Theta B) \oplus B $$

where A is the image to be processed and B is the structural element.

## Results

### Monitoring results

#### Analysis of monitoring results

This article uses python3.10, pycharm2022.2.2, and opencv4.6.0 to process camera data at K15+0.10 km on the G2001 line of the Jinan Ring Expressway.

**Figure 4:**
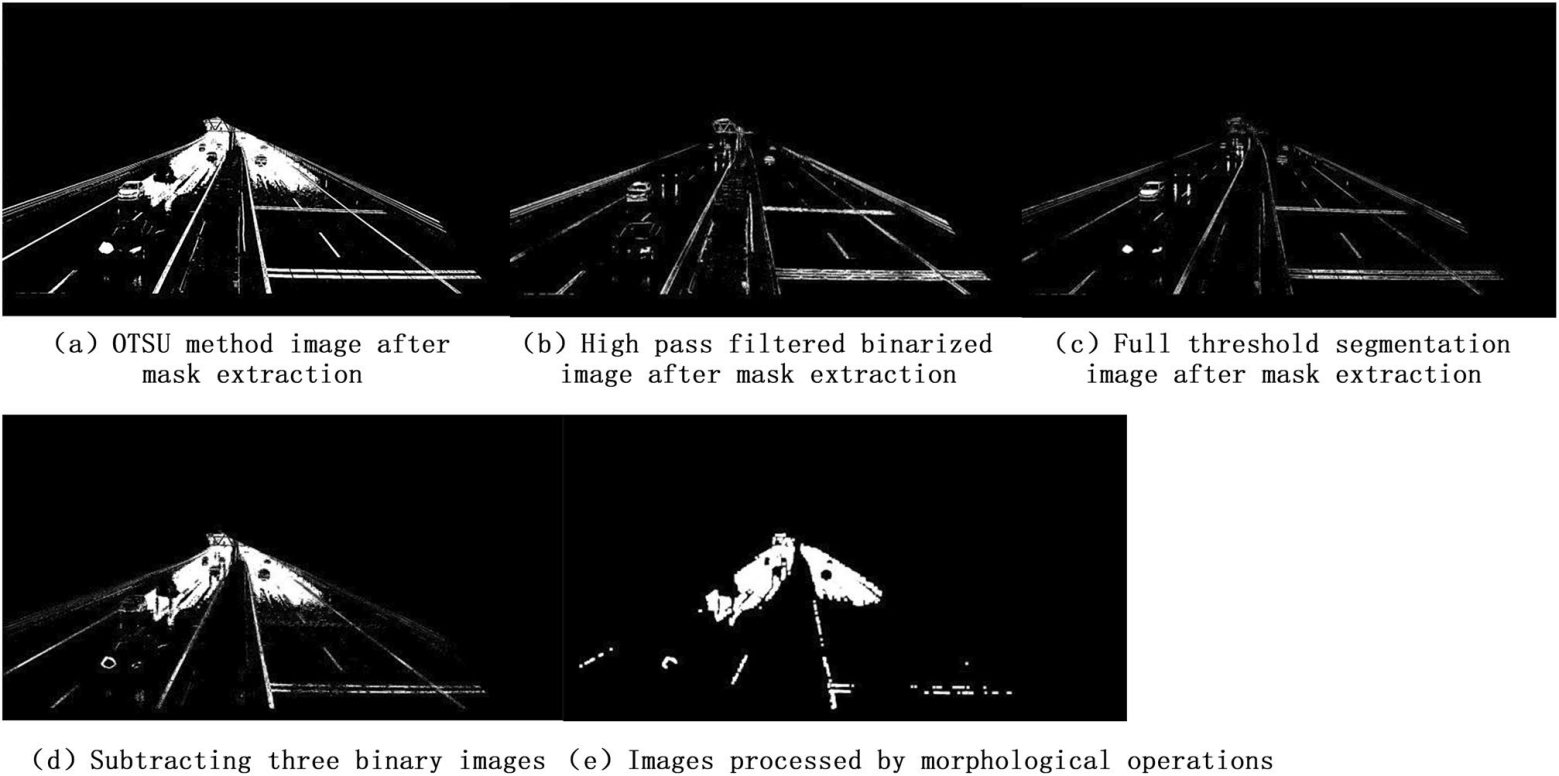
Rain coefficient model processing process. The above figure shows the process of processing images for this model.

The image captured by the camera at 16:10:45 on October 1, 2022 is shown below. The weather at this time was light rainy. The monitoring model in this article was used to process and calculate the image, resulting in a P_rain coefficient_ of 8.72%. The processing process of this model is shown in Fig. 4 below. The images of OTSU method, high-pass filtering, and full threshold segmentation have been shown in Fig. 3, and will not be shown here.

#### Judgment of rain size

The rain coefficient model reflects the amount of water and snow on the road surface by the proportion of white pixel blocks. By calculating different periods and lots, combined with weather forecast predictions, when the P_rain coefficient_ is between 0 and 5%, it is judged as no rain (cloudy); when the P_rain coefficient_ is between 5 and 11%, it is judged as light rain; when the P_rain coefficient_ is between 11 and 20%, it is judged as moderate rain; and when the P_rain coefficient_ is 20% or more, it is judged as heavy rain.

#### The effect of headlights on monitoring results

On rainy days, as the intensity of the rain increases, a driver's visibility will become increasingly obscured, necessitating the use of headlights. The headlights illuminate the road surface, enhancing the reflectivity of the water thereon. This increase in reflectivity, in turn, boosts the overall brightness of the road, leading to a positive correlation between the impact of the lights and the intensity of the rain; that is, the heavier the rain, the brighter the road appears.

### Validation

#### Data verification of cameras at different times on the Jinan Ring Expressway

**Figure 5:**
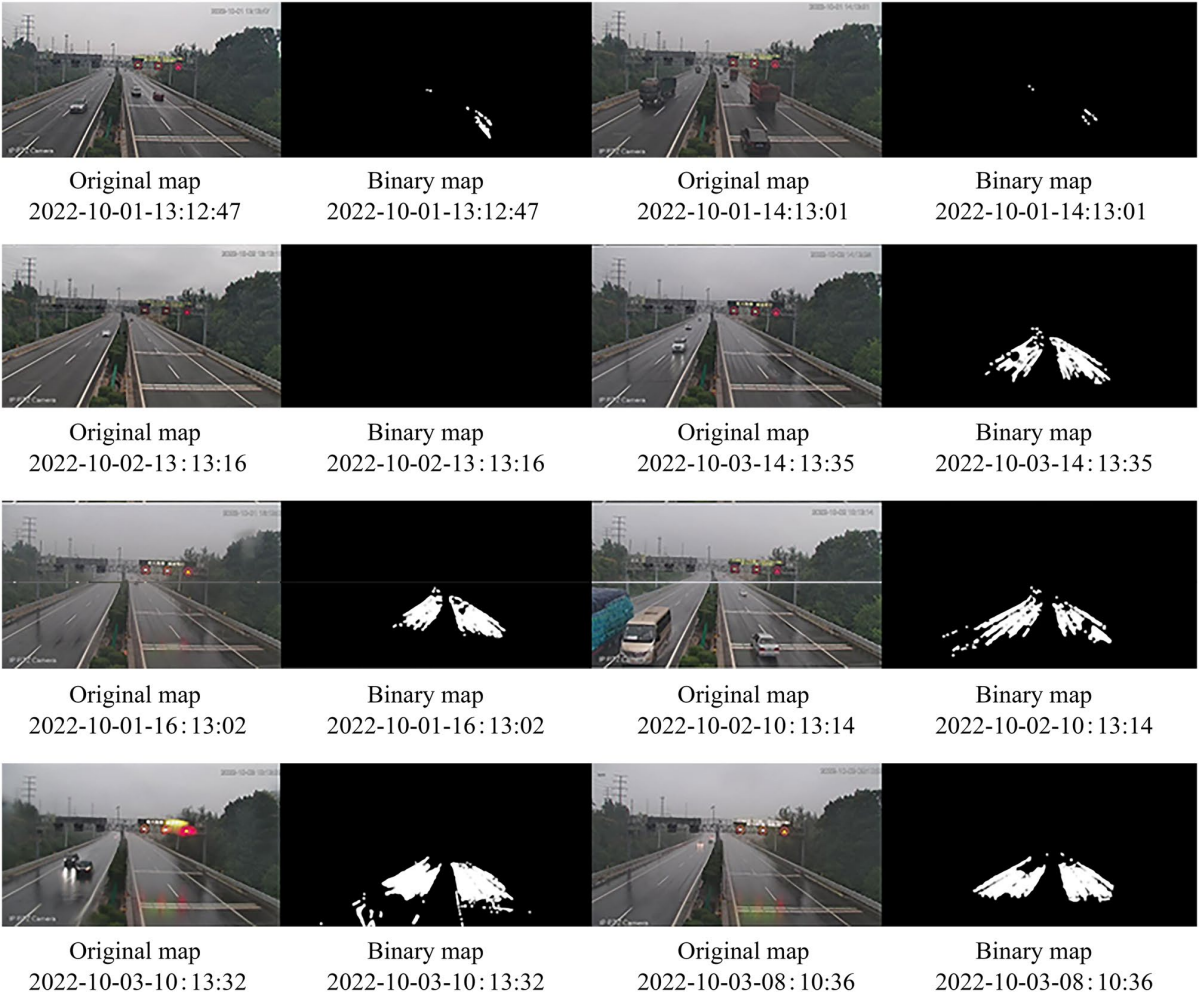
Data validation of Camera at K15+0.10 km of Jinan Ring Expressway G2001 Line in different time periods. The above image shows the original and processed binary images of the camera at K15+0.10 km of Jinan Ring Expressway G2001 at different time periods.

In order to verify the accuracy of the experimental results, the camera at K15+0.10 km of Jinan Ring Expressway G2001 Line at different time intervals is selected for data validation. The verification results are shown in Table 1. The original images and processed binary images of the camera at G2001 K15+0.10 km of Jinan Ring Expressway at different time periods as shown in Fig. 5.

**Table 1:** Camera data at K15+0.10 km of Jinan Bypass Highway G2001 at different times.

Time	Weather	P_rain coefficient_ (%)
2022-10-01-13:12:47	Cloudy	0.68
2022-10-01-14:13:01	Cloudy	0.30
2022-10-02-13:13:16	Cloudy	0.00
2022-10-03-14:13:35	Light rainy	6.85
2022-10-01-16:13:02	Light rainy	6.55
2022-10-02-10: 13:14	Light rainy	8.31
2022-10-03-10:13:32	Medium Rainy	12.78
2022-10-03-08: 10:36	Light rainy	9.21

#### Data validation for other road sections

**Figure 6:**
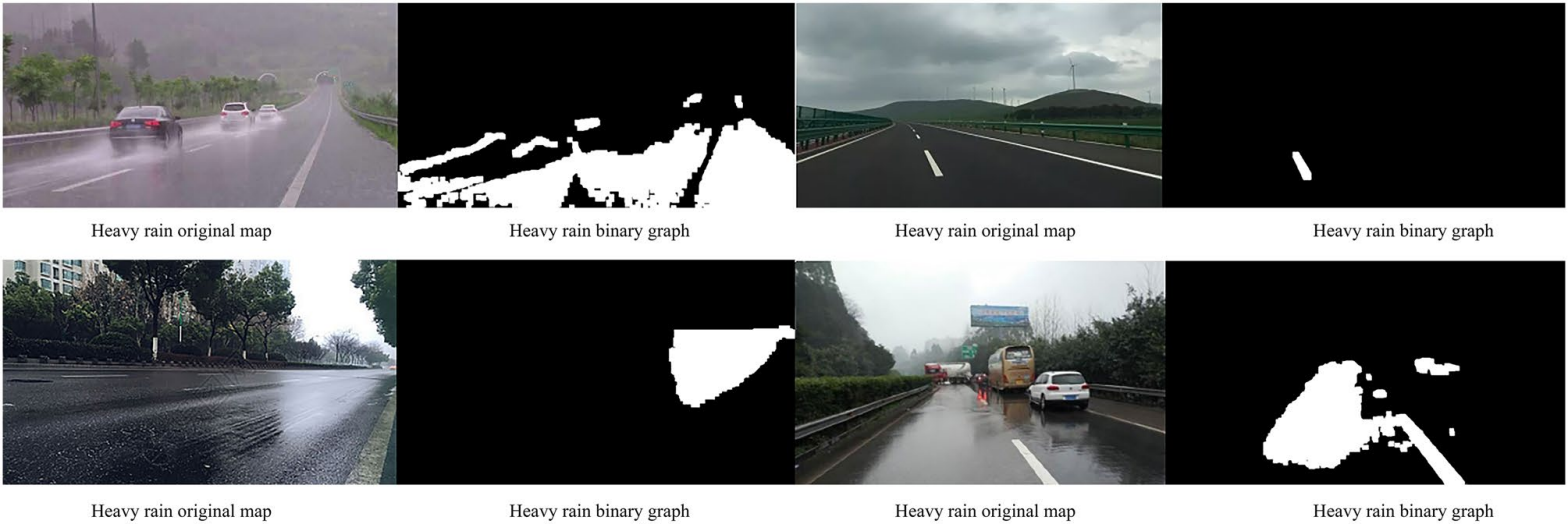
Data validation for other road sections. The above image shows the original and processed binary images of the weather on other road sections.

To verify the universality of the experimental results, other road sections were selected to validate this model, as shown in Table 2. The original and processed binary images of weather on other road sections as shown in Fig. 6.

**Table 2:** Data validation for other road sections.

Weather	P_rain coefficient_ (%)
Heavy rainy	57.57
Cloudy	0.93
Light rainy	8.20
Medium rainy	19.99

## Discussion

In this paper, a series of operations such as high-pass filtering, Otsu method, full domain value segmentation, mask extraction of roads, and morphological open operation is used to construct a rain coefficient model to find out the percentage of roads occupied by standing water and to minimize the influence of vehicles and noise on the roads, thus ensuring the highest possible accuracy.

### Model error analysis

**Figure 7:**
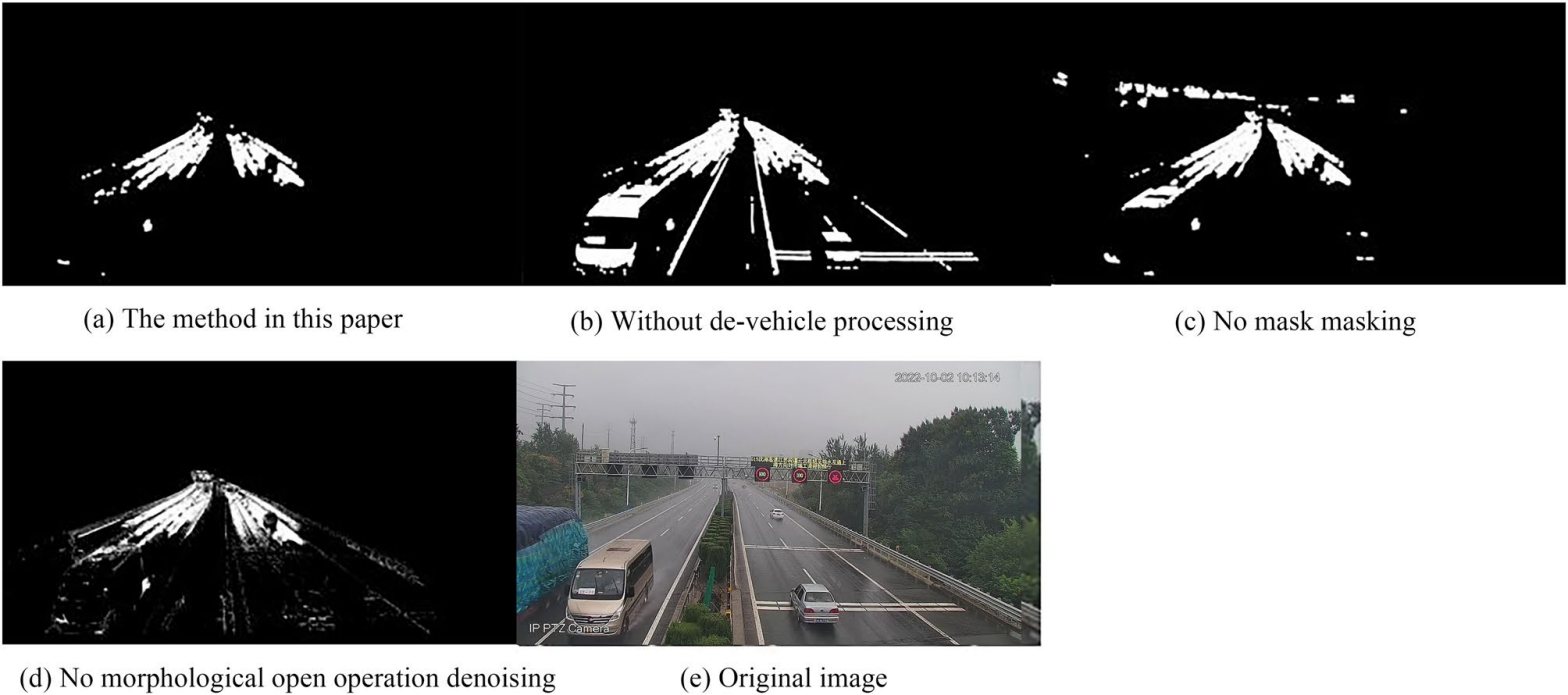
Model error analysis. The above figure shows four sets of comparative images and original images obtained from the model accuracy.

In order to verify the accuracy of the model, the following four images obtained by the method in this paper, without removing vehicles, without performing masking, and without morphological opening denoising are compared. The images used in this paper are the ones taken on October 2, 2022 at 10:13:14 in light rain. Figure 7a is the algorithm constructed by the author, Fig. 7b is the image obtained without removing vehicles, and vehicles have a large impact on the result; The algorithm in this paper is calculated on the road, and masking can extract the road, eliminate the impact of white pixel blocks outside the road on the result, Fig. 7c is the image obtained without performing masking; The algorithm in this paper uses morphological opening operations to remove the impact of noise on the final result, Fig. 7d is the image obtained without performing morphological opening denoising; Fig. 7e is the original image, and the weather is light rainy. Table 3 lists the percentage of water accumulation on the road under four different conditions.

**Table 3:** Percentage of ponded water on the road calculated for the four scenarios.

Algorithm	The algorithms in this paper	No de-vehicle processing	No mask masking	No morphological open operation denoising
P_rain coefficient_	8.31%	21.58%	2.98%	9.81%

### Analysis of monitoring accuracy

In terms of the rain size judgment according to 3.1.2, as well as the comparisons of this paper's algorithm with other algorithms and the weather conditions at that time in 4.1, it can be concluded that the highest accuracy is achieved when applying the method in this paper, and corresponding results can be obtained for the weather conditions at that time. The de-vehicle operation in this paper's algorithm is divided into two steps. The first step extracts the contour line of the vehicle using high-pass filtering, which primarily removes vehicles with higher gray values. The second step performs full-domain value segmentation, which is used to eliminate vehicles with lower gray values to eliminate their impact on the road. The purpose of mask processing is to remove the influence of factors other than the road on the results. The purpose of morphological opening denoising operation is to eliminate the effect of noise on the results. These parts are combined to construct the complete rain coefficient model in this paper.

### Applicability of the model

**Figure 8:**
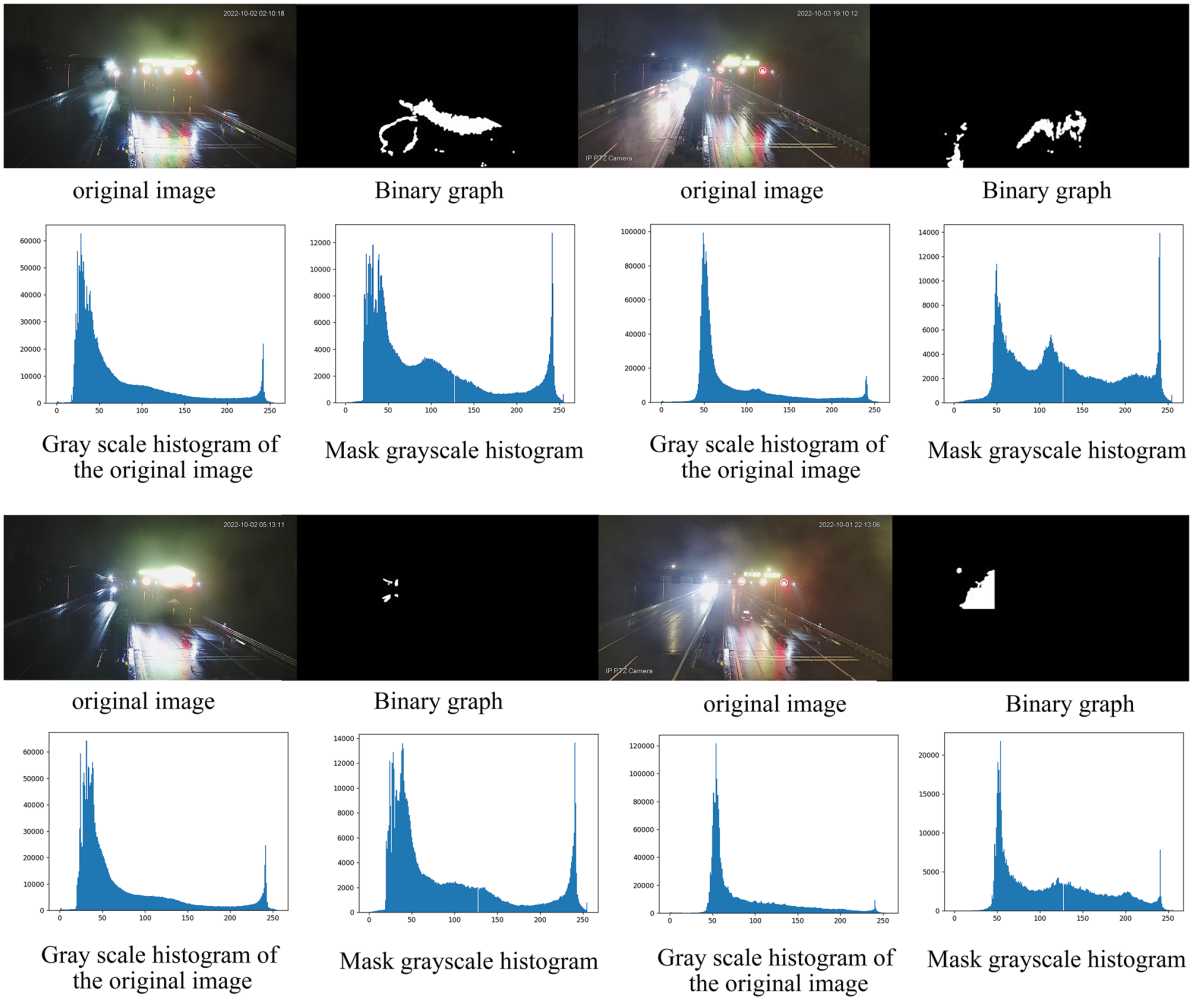
Validation of nighttime data model applicability. The above image shows the validation of the applicability of the night image to the model and the extraction of grayscale histograms before and after the road.

The construction of the monitoring model in this paper is only applicable to daytime rain monitoring. The reason for this is that the OTSU method relies on dividing the image into two parts: background and foreground, based on the distribution of gray values. The dividing value between these two parts represents the threshold value we require. However, at night, the distribution of gray values is concentrated in lower and higher intervals, resulting in a smaller percentage of intermediate gray values. Consequently, the applicability of the model is greatly reduced, leading to a significant decrease in accuracy. As shown in Table 4 and Fig. 8, these limitations are clearly demonstrated.

**Table 4:** Validation of nighttime data model applicability.

Weather	P_rain coefficient_ (%)	Measure the weather
Heavy rainy	6.32	Light rainy
Medium rainy	5.27	Light rainy
Light rainy	1.55	No rainy
Light rainy	14.62	Medium Rainy

## Conclusion

This paper combines high-pass filtering, Otsu method, full domain value segmentation, mask extraction road, morphology open operation, proposed for the road rain monitoring coefficient model P_rain coefficient_ of, according to the value of P_rain coefficient_ to determine the magnitude of the rain and to verify the algorithm in this paper with different sections of Jinan bypass highway G2001 line, different period of the camera sunny, cloudy, rainy weather data, this paper The main findings of the research are.

1. Using the algorithm proposed in this article to detect rainy days, a rain monitoring model P_rain coefficient_ is constructed. The results obtained from the model are compared with the weather forecasted by meteorological forecasts, which can accurately identify cloudy and rainy days, and the magnitude of rainfall can be determined based on the value of P_rain coefficient_. In the result monitoring analysis, at 16:10:45 on October 1, 2022, the image calculated a P_rain coefficient_ of 8.72% for light rain, which was the same as the weather forecasted by meteorological forecasts at that time.
2. This method is not only applicable to expressways, but also to ordinary road sections; however, due to the model being constructed based on grayscale values, there are certain limitations in the model at night, and further research is needed for nighttime monitoring models.
3. To verify the accuracy of the model, the method proposed in this article has the highest accuracy. Using a picture of light rain at 10:13:14 on October 2, 2022, the model in this article was verified. The P_rain coefficient_ = 8.31% without vehicle removal, P_rain coefficient_ = 21.58% without masking, P_rain coefficient_ = 2.98% without morphological opening denoising, and P_rain coefficient_ = 9.81% without morphological opening denoising.
4. This monitoring model is currently only applicable during the daytime. In future research, we will continue to improve this model to make breakthroughs in nighttime monitoring. We will also integrate it with deep learning to improve the deep learning model.

## Data Availability

The datasets used and/or analysed during the current study available from the corresponding author on reasonable request.
